# Factors Associated With Violence Against Children in Low- and Middle-Income Countries: A Systematic Review and Meta-Regression of Nationally Representative Data

**DOI:** 10.1177/1524838020985532

**Published:** 2021-01-19

**Authors:** Ilan Cerna-Turoff, Zuyi Fang, Anne Meierkord, Zezhen Wu, Juan Yanguela, Clare Ahabwe Bangirana, Franziska Meinck

**Affiliations:** 1 Department of Global Health and Development, 4906 London School of Hygiene and Tropical Medicine, London, United Kingdom; 2 Department of Social Policy and Intervention, 6396 University of Oxford, United Kingdom; 3 12211 Faculty of Medicine, University of Southampton, United Kingdom; 4 Department of Applied Psychology, 5894 New York University, USA; 5 AfriChild Centre, 308367 College of Humanities and Social Sciences, Makerere University, Kampala, Uganda; 6 School of Social and Political Science, University of Edinburgh, United Kingdom; 7 Faculty of Health Sciences, North-West University, Vanderbijlpark, South Africa

**Keywords:** child abuse, etiology, prevention of child abuse, domestic violence, adolescent victims, sexual assault

## Abstract

**Background:**

This systematic review and meta-regression sought to identify the relative importance of factors associated with physical, emotional, and sexual violence against children in low- and middle-income countries. Understanding of factors associated with violence is important for targeted programming and prevention on the population level.

**Methods:**

We searched 17 electronic databases from 1989 to 2018 and reports from child violence surveys. Nationally representative studies that described evidence on potential factors associated with violence against children under 18 years old were included. The search was restricted to the English language. Factors were synthesized quantitatively using robust variance estimation, with 95% confidence intervals, for each violence type.

**Results:**

We identified 8,346 unduplicated studies, and 103 publications met our eligibility criteria. The data distribution was uneven across region, country income status, factors, and violence types. Of the 94 eligible studies quantitatively synthesized, no specific factors were significant for physical violence. Lower household socioeconomic status, being a girl, and primary education of mothers and adults in the household were associated with emotional violence, and being a girl was associated with sexual violence.

**Conclusion:**

A broad spectrum of factors merit consideration for physical violence policy and prevention among the general population of children in low- and middle-income countries. Conversely, a tailored approach may be warranted for preventing emotional and sexual violence. Information is unequally distributed across countries, factors, and violence types. Greater emphasis should be placed on collecting representative data on the general population and vulnerable subgroups to achieve national reductions in violence against children.

Violence against children is a recognized global public health issue. Estimates suggest that globally 1 billion children between the ages of 2 and 17 experienced some form of violence in the past year, with the highest prevalence rates in Africa, Asia, and North America where at least 50% of children experienced past year violence (Hillis et al., 2016). Meta-analyses of self-reported cases indicate that approximately 23% of the world’s children experienced lifetime physical abuse, 36.3% experienced emotional abuse, and 13% experienced sexual abuse (Stoltenborgh et al., 2012; Stoltenborgh et al., 2013; Stoltenborgh et al., 2011). An increasing number of studies over the past two decades illustrate that violence in childhood has a relationship with health risk behavior, disease, epigenetic alterations and aging, intergenerational transmission of violence, and an array of other social and health factors (Anda et al., 2006; Dunn et al., 2013; Fulu et al., 2017; Hughes et al., 2017; Jovanovic et al., 2017). Global agreements to protect children from violence have existed since the ratification of the Convention on the Rights of the Child (CRC) and increased in their visibility with publication of the 2002 *World Report on Violence and Health* (Krug et al., 2002; United Nations General Assembly, 1989). The importance of the issue recently has been enshrined in Sustainable Development Goal (SDG) 16.2 which outlines an ambitious target of ending all forms of violence against children by 2030 (United Nations General Assembly, 2015).

Violence prevalence tends to be higher in low- and middle-income countries as compared to high-income countries. By way of example, a national school-based study in the high-income country of Switzerland found that 22% of girls and 8% of boys experienced some form of sexual violence in their lives, whereas implementation of the same survey in the low-income country of South Africa yielded a prevalence rate of 33.9% for girls and 36.8% among boys (Averdijk et al., 2011; Ward et al., 2018). Violence estimates, notwithstanding, vary considerably among countries, and wide differences exist by violence type (physical, emotional, and/or sexual) and by gender (Stoltenborgh et al., 2012; Stoltenborgh et al., 2013; Stoltenborgh et al., 2011). The patterning of violence in low- and middle-income countries, however, indicates that children may experience higher levels of polyvictimization, or the co-occurrence of multiple violence forms, than in high-income countries (Le et al., 2018). When violence forms cluster, it can lead to worse health outcomes throughout the life course (Anda et al., 2006; Felitti et al., 1998).

Low- and middle-income countries further are disproportionately burdened by violence against children. First, a difference exists in demographic profiles. Upward of 80% of the world’s children reside in low- and middle-income countries (United Nations Children’s Fund [UNICEF], 2019b). A larger proportion of the total population is therefore at risk, and violence against children has a greater role in shaping development indicators. The World Health Organization (WHO, 2018) estimates that over 94% of the Disability-Adjusted Life Years lost to interpersonal violence against children, aged 0–15 years, occurred in low- and middle-income countries in 2016. Second, low- and middle-income countries face greater constraints in allocation of their health and social protection budgets. Low- and middle-income countries have limited fiscal resources for national systems strengthening and, in parallel, are stretched by multiple competing priorities in terms of insufficient infrastructure to access children equally within countries; greater instability from armed conflict, natural hazards, and/or widespread political violence; and a dual burden of chronic and infectious disease which overtaxes health systems and promotes unequal investment (Crea et al., 2018; Krueger et al., 2014; Kushitor & Boatemaa, 2018; Peduzzi et al., 2009; Remais et al., 2013; Schleussner et al., 2016). Exact national expenditures on child protection are difficult to calculate or compare across countries because of the lack of uniform data and the diverse number of programs and policies involved in the system (Pereznieto et al., 2011). The International Labour Organization (ILO, 2017), nevertheless, estimates that investment in social protection, excluding health, between 2017 and 2019 was exceptionally limited among most low- and middle-income countries as compared to high-income countries, given the percentage of children within their total populations. The general trend shows that nation-states in Africa spent less than 0.6% of their gross domestic product (GDP) on social protection for children under the age of 14, whereas Northern, Southern, and Western Europe spent on average 2.5% of their GDP (ILO, 2017). Third, systems of social protection are less integrated or robust to prevent violence against children (Crea et al., 2018). A report from the UNICEF (2012) found that 14% of 43 evaluations from low- and middle-income countries incorporated child protection into national and decentralized processes. As a result, low- and middle-income countries present a distinct context and are a priority in reducing global violence against children.

Data coverage on all forms of violence and across all low- and middle-income countries is lacking despite the ramifications for children (UNICEF, 2014). SDG 16.2 has spurred progress in that a greater number of countries are documenting the proportion of children protected from and affected by violence. UNICEF and the WHO, however, found that most low- and middle-income countries either had no data or were not meeting progress targets to achieve the eradication of violence by 2030 (UNICEF, 2019a; WHO, 2020). Low- and middle-income countries that do not have extensive domestic data on violence could benefit from evidence on which factors are most strongly associated with violence for targeting of population-level interventions.

Past literature reviews on child violence risk or associated factors have taken the approach of analyzing studies from individual countries or regions (Meinck, Cluver, Boyes, & Mhlongo, 2015; Spencer et al., 2019) or have applied narrow categories of victimization, such as dating violence, for data synthesis (Hébert et al., 2019). Large-scale studies, such as the Violence Against Children Surveys (VACS), have filled in informational gaps on factors related to violence by collecting nationally representative data across multiple countries and using standardized methodologies and questionnaires which has allowed investigators to pool the data and determine key correlates (Maternowska & Fry, 2018; Palermo et al., 2019; Ravi & Ahluwalia, 2017). A global review and meta-analysis of the literature on the salient population-level factors associated with violence against children in low- and middle-income countries has not been conducted. The purpose of this systematic review and meta-regression is to identify and understand the relative importance of factors associated with physical, emotional, and sexual violence against children in low- and middle-income countries.

## Method

The search strategy followed the *Cochrane Handbook for Systematic Reviews of Interventions* for guidance and the Preferred Reporting Items for Systematic Reviews and Meta-Analyses for reporting (Higgins & Green, 2011; Liberati et al., 2009). The review protocol was prospectively registered in PROSPERO (CRD42017062650).

### Inclusion and Exclusion Criteria

We created standardized inclusion and exclusion criteria prior to screening (refer to Table 1). The research team included studies that described original quantitative research, used representative sampling methods on the national level, and appeared in peer-reviewed journals or gray literature reports. The search was restricted to the English language. We included articles in which children under the age of 18 reported on their own experiences of violence or adults or caregivers reported on violence perpetrated against children. Studies that engaged adults, aged 18 years and older, and asked them to self-report retrospectively on violence experienced in their childhood were excluded to minimize recall bias (Fergusson et al., 2000; Hardt & Rutter, 2004). We additionally excluded articles that reported on administrative data or hospital data which tend to capture a smaller number of cases than found within the general population. Secondary reviews of literature were excluded, and in the case of systematic reviews, bibliographies were hand searched for additional relevant sources.

**Table 1.** Inclusion and Exclusion Criteria.

Inclusion	Exclusion
1. Person experiencing violence is under 18 years old	1. Adult retrospective report of violence experienced as a child
2. Low- or middle-income country at the time of data collection	2. High-income country at the time of data collection
3. Data collected after November 20, 1989	3. Data collected before November 20, 1989
4. Quantitative study	4. Qualitative study
5. Nationally representative sample	5. Nonrepresentative samples or a subnational study
6. Original research published in a gray literature report or peer-reviewed journal	6. Editorials, narrative reviews, or general reports that do not introduce new evidence
7. Risk, protective, or promotive factor associated with violence included	7. Case studies, case control or other matched studies, administrative data or hospital records, conference abstracts, or secondary reviews of literature
8. Physical, emotional, or sexual violence as the outcome measure of the study	8. Gang violence; female genital mutilation; homicide; child labor, exploitation, trafficking, or marriage; neglect; attitudes toward violence; or physical or emotional violence perpetrated by children as outcome

### Search Strategy

We drafted a search strategy to identify original quantitative evidence from peer-reviewed articles and gray literature (refer to Online Appendix A for Medline/PubMed search strategy). Search strings included terms that related to four thematic areas: (a) children; (b) factors associated with violence; (c) physical, emotional, and sexual violence; and (d) low- and middle-income countries. The search terms were adapted from published search strategies on children (Boluyt et al., 2008), risk (Geersing et al., 2012; Shenderovich et al., 2016), violence (Meinck, Cluver, Boyes, & Mhlongo, 2015), and lists of current and past names of low- and middle-income countries (Cochrane Collaboration, 2012; World Bank, n.d.).^1^

Children were defined as anyone below the age of 18 (United Nations General Assembly, 1989). By our definition, associated factors included internationally comparable characteristics or experiences that could feasibly precede violence on the causal pathway. We included risk factors (positively associated with violence) and protective factors (negatively associated with violence) as well as vocabulary related to correlation and statistical association in our search strategy for this review. Risk and protective factors must precede the exposure of interest (Gordis, 2014). Most studies of violence in low- and middle-income countries, however, are cross-sectional (Le et al., 2018; Meinck, Cluver, Boyes, & Mhlongo, 2015; UNICEF, 2014). We applied an inclusive definition to capture as broad a body of literature from these settings as possible. Past experiences of violence were likewise included in analyses when available to acknowledge that past violence can exacerbate future violence risk (Anda et al., 2006; Felitti et al., 1998). We excluded attitudes on violence, given the global cultural variability (Dubowitz et al., 2018). The World Bank (n.d.) country designation at the time of data collection, based on gross national income per capita, was used to define a low-, lower middle-, or upper middle-income country (Fantom & Serajuddin, 2016). We applied physical, emotional, and sexual violence typologies found in UNICEF’s *Hidden in Plain Sight* report to operationalize our definition of violence. In attempting to gain specificity in violence forms and reduce differences in our comparison of factors, we restricted the definition of physical and emotional violence to acts perpetrated by adults against children and sexual violence to acts committed by persons within any age-group (UNICEF, 2014).

### Study Screening

The research team searched 17 electronic databases dating from November 20, 1989 until February 4, 2018, including unindexed articles. We decided upon the final list of included databases in consultation with two information specialists (refer to Online Appendix B for a full list of databases). We selected the beginning of this review as the ratification date of the CRC which was a watershed moment in global child rights that aided in establishing global standardization of the age of childhood as under 18 years (United Nations General Assembly, 1989). The bibliographies of systematic reviews identified in the literature search were examined by a member of the research team for additional eligible studies. In an effort to ensure comprehensive coverage, the research team directly screened gray literature reports pertaining to national-level surveys on violence against children in low- or middle-income countries. These reports include (1) Demographic and Health Surveys (DHS), (2) Multiple Indicator Cluster Surveys (MICS), (3) VACS, (4) Global School-Based Student Health Survey, (5) the Balkan Epidemiological Study on Child Abuse and Neglect, (6) Union Bank of Switzerland (UBS) Optimus violence studies, and (7) Health Behavior in School-Aged Children Survey. We solicited recommendations on additional published and unpublished literature from 22 global violence experts.

Members of the research team conducted a double-blind screening of titles and abstracts after the removal of duplicates, using Rayyan QCRI systematic review management tool (Ouzzani et al., 2016). Independent inclusion decisions were reconciled jointly in consultation with the lead author. The research team subsequently worked in pairs to read full article texts for independent inclusion decisions. Following consistent procedures, the research team reconciled their choices and consulted with the lead author to formalize any outstanding differences. The research team contacted the corresponding authors of included publications if key information could not be deciphered from the text or if the study used a broader age range that exceeded the specifications of this review. Two authors provided further information for inclusion in the final analysis—one author provided a list of sources used for a meta-analysis (Ji & Finkelhor, 2015) and another provided subgroup analysis for children under 18 years old (Meinck et al., 2017).

### Data Extraction and Quality Assessment

The research team independently double extracted information from full texts that met the inclusion criteria. Discrepancies were resolved by consensus after reviewing the original text. The standardized data extraction sheet included information on publication specifics, including the country of data collection, region, and income status; study population; methodological characteristics, such as sample size and study design; named factors; and type of violence outcome. The review team extracted factors by violence type as named by the study authors. All factors, whether significant or insignificant, were extracted. The risk of bias was assessed independently and in duplicate, using the Joanna Briggs Institute checklists for cross-sectional studies (Moola et al., 2017). The risk of bias was not used as the basis for decisions about inclusion or exclusion as per Cochrane Handbook guidelines (Higgins & Green, 2011).

### Data Analysis

Detailed information on the included studies and identified factors for each violence type were summarized in a narrative form. We subsequently synthesized the relationship between factors by violence type, using robust variance estimation. Robust variance estimation is a meta-regression technique that accounts for dependencies among nonindependent effect sizes, so that multiple estimates can be included from each study and incorporates adjustment for study heterogeneity directly as part of calculating variance (Tanner-Smith et al., 2016). The multivariate approach, furthermore, allows for comparison of multiple factors in relation to one another (Hair et al., 2010). Typically, meta-regression is utilized within a meta-analysis for conducting moderator or subgroup analyses of the pooled effect estimate (Borenstein et al., 2009). In this analysis, we are interested in the relative values of factors, as indicated by the β coefficients, which contribute to physical, emotional, and sexual violence rather than the pooled effect estimates for violence outcomes. Robust variance estimation is particularly appropriate when estimating fixed effects like β coefficients and when analyzing multiple measures from studies that sample overlapping populations (Tanner-Smith et al., 2016). We capitalized on these aspects of our meta-regression to identify the relationships among all possible factors for each violence outcome on the population level.

The meta-regression conformed to a predetermined plan for pooling identified factors. We first sorted factors into consistent and comparable categories for analysis and divided by violence type. A minimum of 10 estimates was set as the threshold per covariate for inclusion in the meta-regression. No set cutoff exists on the minimum number of estimates needed per study or per covariate when robust variance estimation uses small sample corrections. We, therefore, followed general guidance in making modeling and reporting choices that reflected a balance between the number of studies and average number of estimates per study (Tanner-Smith et al., 2016). Prevalence measures or effect estimates subsequently were calculated and converted to a common scale as log odds ratios when possible. Prior to synthesis, we inspected the distribution of the data in violin plots and the presence of potential outliers in boxplots. A conservative rule of thumb of 2.5 standard scores from the remaining data was utilized for determining outliers (Borenstein et al., 2009). We then pooled estimates into three models for physical, emotional, and sexual violence. The factors were synthesized using robust variance estimation, with 95% confidence intervals (CIs), correlated effects weights, and small sample corrections. Models were assessed before and after winsorizing extreme values to determine the influence of outliers on subsequent pooled models. Significance of covariates in the analysis correspondingly were deemed reliable if containing four degrees of freedom or greater, as indicated from prior simulation studies (Tipton, 2015).^2^ Omnibus *F* tests lastly were conducted to determine the possible presence of joint covariance, indicating additional synergies across factors to influence violence outcomes. Publication bias was examined in *p* value plots to indicate whether studies fell within the extreme upper or lower tails of the distribution of effect estimates in which publication probability is expected to change in favor of positive studies. The data were analyzed using the Robumeta package, and publication bias was assessed using the PublicationBias package in R v.3.3.3 (Fisher & Tipton, 2015; Mathur & VanderWeele, 2020; R Core Team, 2017).

## Results

### Data Description

**Figure 1.**
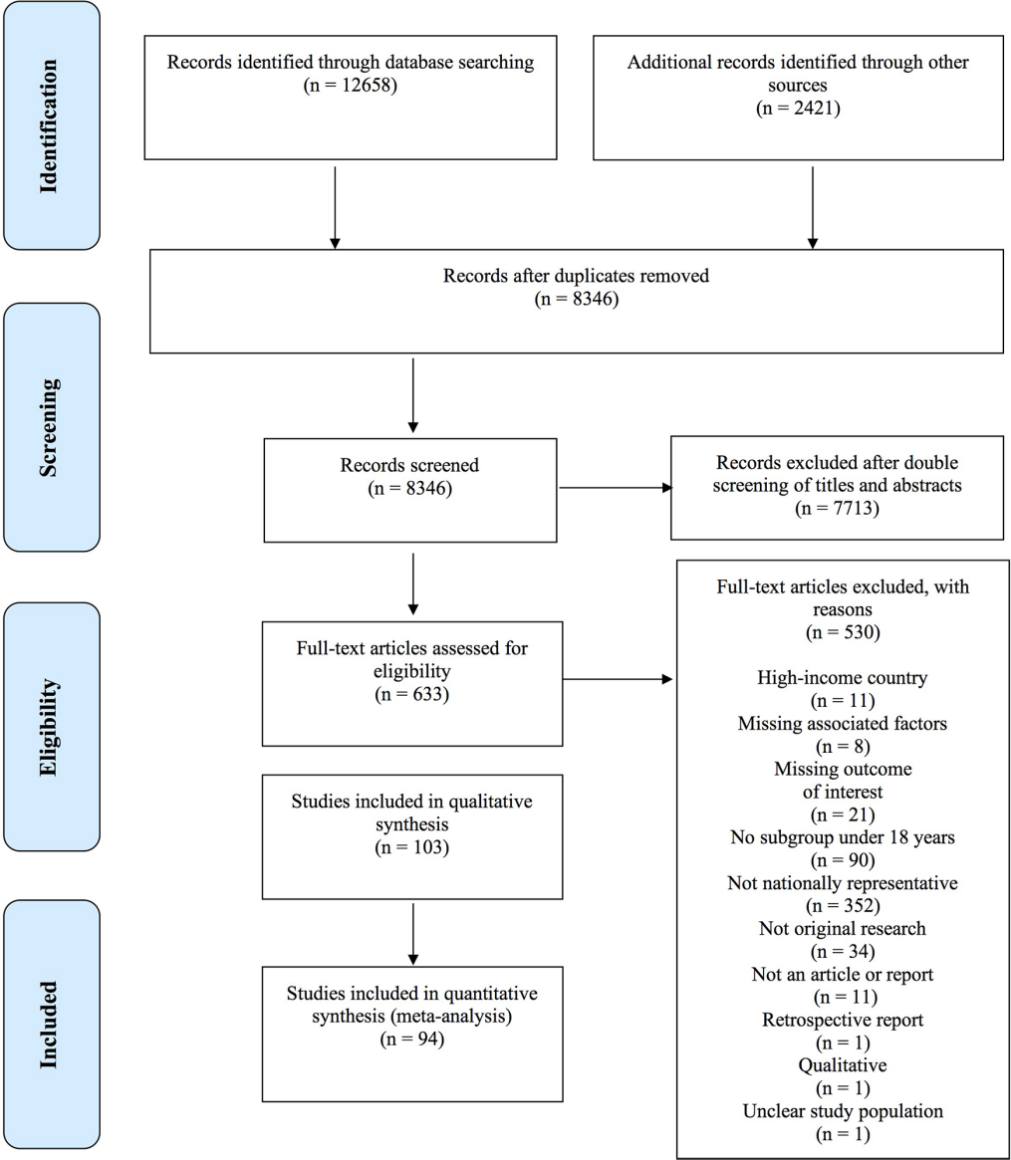
Preferred Reporting Items for Systematic Reviews and Meta-Analyses flowchart. *Source*. Adapted from Moher et al. (2009).

The screening of electronic databases and gray literature reports, expert recommendations, and hand screening of systematic reviews yielded 103 eligible studies (refer to Figure 1).

**Figure 2.**
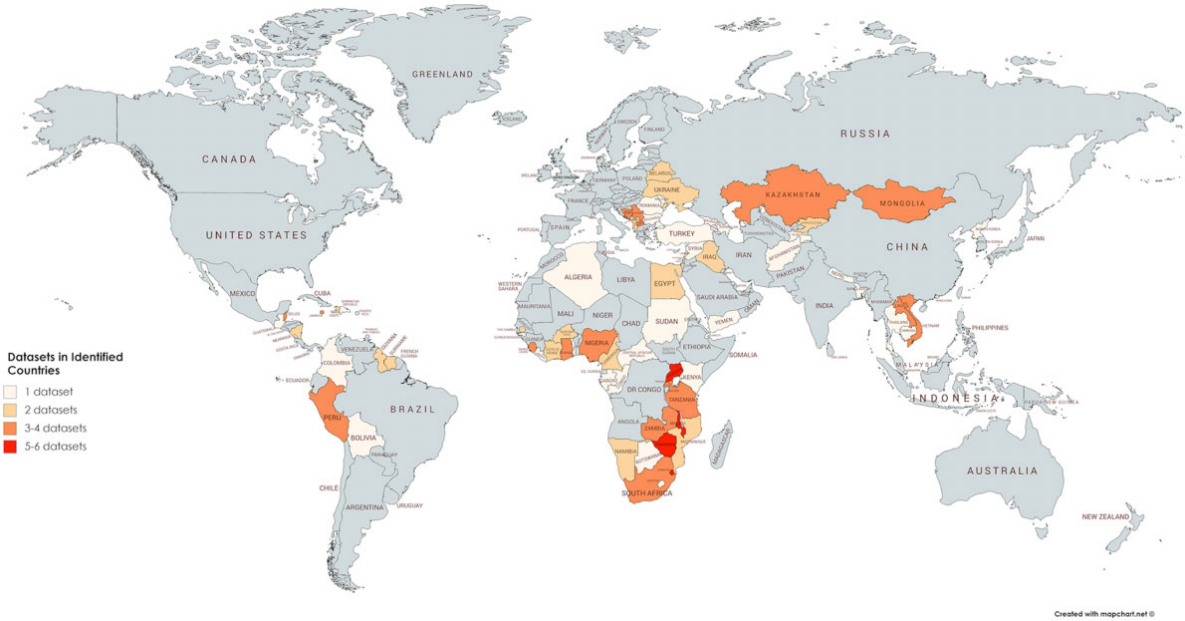
Map of the number of included data sets per country. *Note*. Two countries are not visible on the map—Saint Lucia and Sao Tome and Principe. Saint Lucia is represented in two data sets (Basilyous & Durgampudi, 2016; Ministry of Social Transformation & Local Government and Community Empowerment and Central Statistics Office, 2014), and Sao Tome and Principe is represented in one data set (National Institute of Statistics, 2016).

A total of 151 unique data sets from 75 countries formed the basis of the analysis. The MICS were the most common source of data, followed by the DHS. The distribution of research globally and the number of surveys per country varied vastly. The Africa region returned the largest number of studies with 68 in total, while the region with the least studies was the Southeast Asia region with a total of four studies. Some countries, such as Uganda, were overrepresented, contributing six surveys to the sample while other countries like the Philippines contributed no studies (refer to Figure 2). The distribution of studies across country income levels likewise was uneven. Data mainly were collected in 45 lower- and upper-middle income countries, with slightly fewer studies conducted in 30 low-income countries (refer to Table 2).

**Table 2.** Descriptive Overview of Studies.

Data Characteristics	Number of Countries	Number of Data Sets
World Health Organization region		
African	28	68
Americas	12	23
Eastern Mediterranean	9	12
European	18	34
Southeast Asia	4	4
Western Pacific	4	10
Country income level		
Upper middle	42	35
Lower middle	35	55
Low	18	60
Data source		
AIDS Impact Survey	1	1
Balkan Epidemiological Study on Child Abuse and Neglect	7	7
Caribbean Youth Health Survey	1	1
Demographic Family and Health Survey	1	1
Demographic and Health Surveys	22	27
School-Based Student Health Survey	5	5
Jamaican Youth Risk and Resiliency Behaviour Survey	1	1
Multiple Indicator Cluster Surveys	49	79
National Maternal and Child Health Survey	1	1
National Survey of Social Relations	1	1
Union Bank of Switzerland Optimus	1	1
Other national surveys	14	2
SASA!	1	1
Violence Against Children Surveys	11	11

*Note.* Several countries changed their income level status at specific time points and are documented as separate entries within the table. AIDS refers to Acquired Immunodeficiency Syndrome. SASA! is a Kiswahili word that means “now.”

Nearly all of the studies estimated violence for the general population of children in households or schools, except for 17 studies—10 studies reported nationally representative estimates disaggregated by gender (Andersson et al., 2012; Artz et al., 2016; Brown et al., 2009; Centers for Disease Control and Prevention, Interuniversity Institute for Research and Development, & Comité de Coordination, 2014; Fry et al., 2016; Kidman & Palermo, 2016; Meinck et al., 2017; Ministry of Gender Labour and Social Development, 2015; UNICEF, Centers for Disease Control and Prevention, & Kenya National Bureau of Statistics, 2012; UNICEF, Centers for Disease Control and Prevention, & Muhimbili University of Health and Allied Sciences, 2011), one study presented national data on children with disabilities (Hendricks et al., 2014), and six studies described factors related to violence for Roma and affiliated ethnic minority groups on the national level (Kosovo Agency of Statistics, 2014; Ministry for Human Rights and Refugees of Bosnia and Herzegovina and the Agency for Statistics of Bosnia and Herzegovina, 2013; Ministry of Health, Ministry of Education and Science, & Ministry of Labour and Social Policy of the Government of Republic of Macedonia, 2012; Statistical Office of Montenegro and United Nations Children’s Fund, 2014; Statistical Office of the Republic of Serbia, 2011; Statistical Office of the Republic of Serbia & United Nations Children’s Fund, 2014). Physical violence was the most commonly described form of violence in 93 studies, and sexual violence was the least often reported in 19 studies. Each article contained information on multiple factors related to violence. The identified factors frequently overlapped between physical, emotional, and sexual violence. The literature outlined 57 potential factors for physical violence, 41 potential factors for emotional violence, and 30 potential factors for sexual violence, of which a total of 42 overlapped and 70 were distinct (refer to Online Appendix C). The overall body of literature largely captured similar factors for each violence form, but the same factors were not measured in all individual studies. Demographic characteristics of age, geographic residence, gender, wealth quintiles of the households, and education level of mothers and other adults in the household were the most commonly reported factors in individual studies. Other factors related to violence—such as community or school level safety, parental substance use, and children’s trading of sex for goods or money—were found in isolated studies.

The quality scores of the included studies ranged from 1 to 8 and had an average score of 5. One of the most important axes of bias pertinent to this review was the standardized measurement of factors related to violence. We found that the vast majority of studies (99/103) ranked highly in this regard. In contrast, 94 studies were ranked lowly as they did not adjust for confounding and did not use valid and reliable violence outcome measures.

### Meta-Regression

A total of 94 eligible studies comprising 2,121 estimates and a sample size of 30,468,296 people were identified for further analysis. Most factors did not meet the threshold of 10 estimates necessary for inclusion in the meta-regression model for each violence form (refer to Online Appendix C). The model for physical violence contained 12 factors that met the threshold for inclusion, and no factor was significant. The omnibus *F* test was slightly insignificant which indicates that the presence of joint covariance was unlikely (*F* = 2.65, *df* = 12.7, *p* = .051). The model for emotional violence consisted of 10 factors. Several factors were significant, namely, the first three quintiles for the economic status of the household, being a girl, and primary education of an adult in the household or the child’s mother. Each of these factors had a positive association with violence, indicating that a factor increased by one unit, holding other factors constant, violence increased by the β coefficient value. For example, after taking into account multiple factors related to emotional violence, increases of household income among the poorest of the poor are significantly associated with slight increases in emotional violence (*b* = .19, 95% CI [0.08, 0.30]). The largest increase in emotional violence was a result of being a girl. These factors likely covary which leads to an additional influence on the emotional violence outcome (*F* = 12.2, *df* = 41.2, *p* < .001). Four factors were eligible for inclusion in the model for sexual violence, but only two factors could be synthesized due to the large variance in measures of parental absence of mothers and fathers, leading to unstable weights. The child’s gender was significant which indicates that being a girl was associated with increased sexual violence victimization. Being a girl and a single or double orphan significantly covaried but were unreliable due to underpowering (*F* = 11,417, *df* = 2.74, *p* < .001). A full table of results for each violence outcome is contained in Table 3. Plotting of each meta-regression for potential publication bias indicated that all studies fell within a reasonable range of publication probability, and the results did not favor positive studies (refer to Online Appendix D).

**Table 3.** Table of Associated Factors by Violence Type.

Violence Types	Physical (*n* = 1,225)			Emotional (*n* = 604)			Sexual (*n* = 206)		
	*b*	95% CI	*df*	*b*	95% CI	*df*	*b*	95% CI	*df*
Household wealth									
Quintile 1—Poorest	−.01	[−0.53, 0.54]	1.58	.19**	[0.08, 0.30]	61.48	—	—	—
Quintile 2—Second	−.06	[−0.56, 0.44]	1.58	.15**	[0.07, 0.23]	60.34	—	—	—
Quintile 3—Middle	−.11	[−0.61, 0.38]	1.58	.14**	[0.06, 0.21]	61.35	—	—	—
Quintile 4—Fourth	−.20	[−0.69, 0.29]	1.57	.06	[−0.00, 0.13]	60.99	—	—	—
Child labor									
Yes	−.06	[−0.56, 0.44]	1.58	—	—	—	—	—	—
Child gender									
Girls	−.39	[−1.16, 0.38]	1.26	.77**	[0.53, 1.00]	4.61	1.00**	[0.99, 1.02]	4.94
Education									
None—Adult	−.24	[−0.72, 0.23]	1.80	.11	[−0.00, 0.23]	44.73	—	—	—
None—Mother	−.20	[−0.70, 0.30]	2.15	.01	[−0.16, 0.19]	12.94	—	—	—
Primary—Adult	−.21	[−0.70, 0.27]	1.78	.14*	[0.01, 0.27]	49.81	—	—	—
Primary—Mother	−.18	[−0.63, 0.27]	1.95	.19*	[0.04, 0.34]	24.81	—	—	—
Intimate partner violence									
Experienced by mother	−.18	[−0.53, 0.17]	2.06	—	—	—	—	—	—
Residence									
Rural	−.27	[−0.80, 0.26]	1.48	.03	[−0.05, 0.11]	47.62	—	—	—
Orphan status									
Single or double	—	—	—	—	—	—	−0.01	[−1.22, 1.19]	1.78

*Note.* Estimates and CIs are rounded to two decimal places. Reference groups: (1) Wealth—Quintile 5—Richest; (2) Child labor—No child labor; (3) Gender—Boys; (4) Education—Tertiary or higher; (5) Intimate partner violence—Not experience by mother; (6) Residence—Urban; (7) Orphan status—Not an orphan. *n* = number of estimates; CI = confidence interval.

*p < .05. **p < .01.

## Discussion

In this review, we sought to examine internationally comparable factors for violence against children and determine their relative associations. The research team identified that six factors—the first three quintiles for economic status of the household, being a girl, and primary education of an adult in the household or the child’s mother—covaried and were significantly associated with increases in emotional violence, and being a girl was associated with large increases in sexual violence. No singular or group of factors were associated with physical violence. Our results are both complementary and distinct from comparable studies of factors associated with violence against children. The identified factors correspond to possible risk and protective factors listed in a past systematic review of the Africa region (Meinck, Cluver, Boyes, & Mhlongo, 2015). In contrast, multivariate analyses across countries have yielded slightly different findings. A recent analysis of VACS data from six countries found that increasing age, nonresidence with a biological father, and school enrollment in some instances were associated with increases in physical, emotional, or sexual violence. Household wealth, however, were inconsistently associated with violence against children (Palermo et al., 2019). A previous four-country study that modeled data from the VACS found that richer households, education attainment, and children in marriage-like relationships had the highest odds of violence (Ravi & Ahluwalia, 2017). These differences in findings highlight how factors associated with violence can differ, even when comparing the same survey across a slightly different set of countries.

A threat to generalizability results from unequal distribution of information. Our review found that information was not equally collected across all violence types and countries. In particular, fewer nationally representative studies collected data on the factors that influence sexual violence against children. A growing number of national violence surveys, principally the VACS or UBS Optimus violence studies, have a strong focus on sexual violence (Artz et al., 2016; Chiang et al., 2016). Nevertheless, the most numerous nationally representative data source on violence against children is the MICS and DHS which are designed to ask caregivers about their child-rearing practices in terms of physical and emotional violence (UNICEF, 2014). The gap underscores the importance of wider coverage and a greater number of nationally representative studies on sexual violence. Sexual violence is stigmatized in most societies, and a lack of equal documentation risks further invisibility and improper programmatic and policy responses. Gaps in information should be identified and supplemented with periodic surveillance via national systems or cross-sectional surveys, such as the VACS (Chiang et al., 2016). We likewise would encourage researchers to continue to harmonize their metrics for measuring factors related to violence against children with those utilized in nationally representative surveys to ensure that data can be broadly cross-compared and specifically to ensure that data are collected for potential factors associated with physical, emotional, and sexual violence individually by violence type and jointly for children whom experience more than one form of violence.

The review identified multiple countries that do not have national-level data on factors related to violence against children. This gap in information extends to several of the world’s most populous countries, such as China, India, and Indonesia. The geographic size and large population of these countries pose a challenge in collecting representative data. National-level data on these countries are nevertheless vital (WHO, 2020). It is further crucial that data are equally represented across nations and regions to have accurate and generalizable information. National-level research on the extent, characteristics of, and factors associated with violence against children is a baseline for the establishment of health and spending priorities and feeds into larger global goals of accelerating strategies to prevent violence against children. National governments are the responsible parties for achieving global commitments to end violence against children within their borders, and national-level data present a logical level of analysis for information on the general population (United Nations General Assembly, 1989, 2015). We know, however, that vulnerable subgroups of children often have a higher prevalence of violence and likely face different factors that increase their risk of violence (Gilbert et al., 2018; Jones et al., 2012; Kidman & Palermo, 2016; Russell et al., 2014). An expansion of nationally representative information is needed on specific factors associated with violence against children with disabilities, ethnic and sexual minorities, and by gender, among others. Donors and policymakers are well-poised to work together in identifying these gaps and in financing and implementing nationally representative studies for both the general population and pertinent subgroups of vulnerable children.

Our methodological approach allows for direct comparison of the factors associated with violence in relation to one another which is useful in identifying population-level prevention efforts. The *World Report on Violence and Health* in 2002 underscored that investments in policy and programming tended to be short-term and reactionary without public health approaches that targeted the underlying drivers of violence (Krug et al., 2002). Since then, violence has become more central issue in international development and investment (United Nations General Assembly, 2015). Policymakers are still faced with a dilemma in understanding how to invest, particularly in low- and middle-income countries where funds are limited, and competing priorities exist. Our analysis identified that no factors were significant for physical violence which may imply that a broad array of characteristics and experiences should be considered in violence prevention efforts within the general population of children in low- and middle-income countries. In contrast, a tailored approach that jointly prioritizes economic development, gender equality, and educational attainment for caregivers may be merited for particular consideration in preventing emotional violence against children. Accordingly, shifting social norms and cultures of impunity is essential in reducing sexual violence against girls on the population level. We do not interpret these findings as all-encompassing or static. The data in this analysis target the general population. While helpful to guide policy, an approach that simply adopts these findings likely would fail to prevent violence against specific groups of vulnerable children, undermining targets for overall violence reduction. We strongly advocate a multitiered approach that seeks to use evidence to define policy for different segments of the population. Policymakers additionally should seek to understand nuances of their own national contexts that lead to violence and to update approaches as a greater number of nationally representative studies are conducted and further associated factors for each violence type are documented. Unique constellations of characteristics and experiences exist on the individual level to increase a child’s risk of violence. The factors identified in this analysis should not be construed as markers for determining violence risk for a specific child, but rather, which factors on the population level would most reduce violence against children (Lipsey & Wilson, 2000) (refer to Table 4).

**Table 4.** Summary Table of Critical Findings.

Critical Findings
National-level data on violence against children are unevenly distributed across countries, factors, and violence types Sexual violence was the least studied form of violence in this review No single factor or group of factors are significantly associated with physical violence within the general population of children in low- and middle-income countries Lower household socioeconomic status, being a girl, and primary education of mothers and adults in the household are significant and covarying factors associated with emotional violence at the national level in low- and middle-income countries Being a girl is associated with large increases in sexual violence at the national level in low- and middle-income countries

### Strengths and Limitations

This review is one of the most comprehensive analyses of factors associated with violence within low- and middle-income countries to date. Most quantitative studies of factors associated with violence typically have restricted their analysis to one violence data set so as to compare measures across countries or have relied upon a narrative synthesis (Meinck, Cluver, Boyes, & Mhlongo, 2015; Meinck, Cluver, Boyes, & Ndhlovu, 2015; Meinck et al., 2017; Palermo et al., 2019; Ravi & Ahluwalia, 2017). Our review attempted to compare an extensive body of evidence across studies from a greater segment of the world’s low- and middle-income countries. We searched a multitude of social science and health databases, using comprehensive search terms to achieve the highest possible sensitivity in detecting relevant studies. In addition, the data were supplemented with a review of prominent gray literature reports on violence, hand searches of systematic reviews, and solicitations from experts. All information was doubly extracted and independently assessed to reduce errors. We synthesized the data using a high-quality method that did not pose the risk of second-order sampling bias by including all relevant estimates in each study. Our methodology permitted cross-comparison of factors associated with violence and produced information of the relative importance of salient factors that may increase the risk of violence among the general population in low- and middle-income countries.

Notwithstanding, the review has several limitations. First, some studies may have been missed in our search strategy if children under the age of 18 were categorized as adults. Studies that examine violence against women, for instance, frequently collect data with “women of reproductive age” which includes a demographic of individuals between 15 and 49 years, and data on the age-group of 15- to 17-year-olds is frequently not available (García-Moreno et al., 2005). Second, violence, if described solely in terms of a behavioral act, may have been missed in the controlled vocabulary search. The risk of missing studies is likely low, given the breadth of violence terms utilized in the search strategy and number of data repositories examined. Relatedly, we did not examine physical and emotional violence among children, as these phenomena transgress the borders between violence against children, bullying, and dating or intimate partner violence. A more conservative and restrictive definition of violence in this case likely yielded greater precision in understanding associated factors with specific violence forms. Fourth, language presented a limitation. A recent review of search strategies for risk factors for child violence in low- and middle-income countries indicated that 85% of relevant articles were in English and 15% were in Chinese, Spanish, Portuguese, Russian, or French. The non-English articles were mainly found within regional databases (Shenderovich et al., 2016). We included several regional databases to counterbalance possible exclusions, but we can assume that a minority of sources were published in a language other than English. Fifth, robust variance estimation adjusts for heterogeneity directly in calculation, so it does not provide all useful information that might be desired in a meta-analytic method, such as indicators of heterogeneity like *I^2^*. Our primary goal was to identify and understand the relative values of the β coefficients for each form of violence, however, which is in-line with the recommended uses for this methodology (Tanner-Smith et al., 2016). Sixth, we identified associated factors for physical, emotional, and sexual violence as distinct outcomes. Violence forms additionally tend to co-occur in the same individuals, and polyvictimization may be more common in low- and middle-income countries (Felitti et al., 1998; Le et al., 2018). It is plausible that the factors associated with individual forms of violence are different from those associated with combinations of two or more violence outcomes. The identification of possible unique factors would have required a factorial statistical design to explore all possible combinations of violence which was beyond the scope of this review. We would encourage further research on this topic in the future to develop a more nuanced understanding of factors associated with violence. Last, our decision to set a minimum threshold of 10 estimates per covariate for inclusion in the meta-regression models excluded many factors identified in the studies from quantitative synthesis. We choose to prioritize precision in constructing the best models possible using the available information, but it may be determined that additional factors are significant as further data are collected in the future.

## Conclusion

We need evidence to guide investment and to achieve the bold goals set out by the SDGs. The overarching objective of this review was to understand the relative importance of factors associated with violence among the general population in low- and middle-income countries. The findings present an evidence-based road map for informing national strategy for the general population and should be integrated with specific evidence on subgroups of vulnerable children to create a multitiered policy platform.

### Implications for Practice, Policy, and Research

- Research should ensure that evidence is balanced across countries, factors, and violence types.
- Understanding the relative importance and interrelationship among factors associated with violence against children allows for better tailoring and prioritization in policy and programming for violence prevention.
- Policymakers should consider a broad spectrum of factors in strategies to prevent physical violence among the general population of children, whereas emotional and sexual violence may merit a tailored approach.
- An evidence-driven and multitiered approach for both the general population of children and subgroups of vulnerable children is important in achieving country commitments toward the 2030 SDGs.

## Supplemental Material

Supplemental Material, Appendix_A_clean - Factors Associated With Violence Against Children in Low- and Middle-Income Countries: A Systematic Review and Meta-Regression of Nationally Representative Data Supplemental Material, Appendix_A_clean for Factors Associated With Violence Against Children in Low- and Middle-Income Countries: A Systematic Review and Meta-Regression of Nationally Representative Data by Ilan Cerna-Turoff, Zuyi Fang, Anne Meierkord, Zezhen Wu, Juan Yanguela, Clare Ahabwe Bangirana and Franziska Meinck in Trauma, Violence, & Abuse

Supplemental Material, Appendix_B - Factors Associated With Violence Against Children in Low- and Middle-Income Countries: A Systematic Review and Meta-Regression of Nationally Representative Data Supplemental Material, Appendix_B for Factors Associated With Violence Against Children in Low- and Middle-Income Countries: A Systematic Review and Meta-Regression of Nationally Representative Data by Ilan Cerna-Turoff, Zuyi Fang, Anne Meierkord, Zezhen Wu, Juan Yanguela, Clare Ahabwe Bangirana and Franziska Meinck in Trauma, Violence, & Abuse

Supplemental Material, Appendix_C - Factors Associated With Violence Against Children in Low- and Middle-Income Countries: A Systematic Review and Meta-Regression of Nationally Representative Data Supplemental Material, Appendix_C for Factors Associated With Violence Against Children in Low- and Middle-Income Countries: A Systematic Review and Meta-Regression of Nationally Representative Data by Ilan Cerna-Turoff, Zuyi Fang, Anne Meierkord, Zezhen Wu, Juan Yanguela, Clare Ahabwe Bangirana and Franziska Meinck in Trauma, Violence, & Abuse

Supplemental Material, Appendix_D - Factors Associated With Violence Against Children in Low- and Middle-Income Countries: A Systematic Review and Meta-Regression of Nationally Representative Data Supplemental Material, Appendix_D for Factors Associated With Violence Against Children in Low- and Middle-Income Countries: A Systematic Review and Meta-Regression of Nationally Representative Data by Ilan Cerna-Turoff, Zuyi Fang, Anne Meierkord, Zezhen Wu, Juan Yanguela, Clare Ahabwe Bangirana and Franziska Meinck in Trauma, Violence, & Abuse

## Notes

1. I.C.T. piloted the search strategy with six articles in the Yale MeSH Analyzer to ensure comprehensiveness (Grossetta & Wang, 2018).

2. The degrees of freedom in robust variance estimation depend upon both the number of studies and features of each covariate (i.e., skew and balance) unlike typical meta-regression techniques. As a result, the degrees of freedom are often surprisingly much smaller than the number of studies. On a practical level, the algorithm will automatically weight degrees of freedom to approximate normality for a *t*-distribution when using small sample corrected hypothesis tests. The degrees of freedom produced by the algorithm should be used in determination of significance of a covariate (Tanner-Smith et al., 2016).
