# Supplementary material for: Factors Associated With Violence Against Children in Low- and Middle-Income Countries: A Systematic Review and Meta-Regression of Nationally Representative Data
Source: Trauma Violence Abuse. 2021 Jan 19;22(2):219–32. doi: 10.1177/1524838020985532 (PMC7961628; doi:10.1177/1524838020985532)
Supplement: Supplemental Material, Appendix_A_clean - Factors Associated With Violence Against Children in Low- and Middle-Income Countries: A Systematic Review and Meta-Regression of Nationally Representative Data [file Appendix_A_clean.pdf]

Appendix A. Medline/PubMed search strategy

Search Number	Search	Results
**Children**		
#1	child*.ab,ti.	1191902
#2	minor*.ab,ti.	255050
#3	schoolchild*.ab,ti.	12308
#4	infan*.ab,ti.	385653
#5	adolescen*.ab,ti.	224123
#6	newborn*.ab,ti.	145148
#7	preschool*.ab,ti.	23520
#8	pre-school*.ab,ti.	4325
#9	kindergarten*.ab,ti.	5248
#10	underage.ab,ti.	919
#11	youth.ab,ti.	48564
#12	youths.ab,ti.	9661
#13	baby.ab,ti.	33012
#14	babies.ab,ti.	32689
#15	prepubescen*.ab,ti.	902
#16	pubescen*.ab,ti.	1939
#17	schoolage.ab,ti.	40
#18	school-age.ab,ti.	11183
#19	boy*.ab,ti.	134984
#20	girl*.ab,ti.	129313

#21	offspring.ab,ti.	59539
#22	pediatric*.ab,ti.	234505
#23	paediatric*.ab,ti.	51927
#24	juvenile*.ab,ti.	70452
#25	toddler*.ab,ti.	8427
#26	"nursery school*".ab,ti.	1013
#27	"elementary school*".ab,ti.	8215
#28	"high school*".ab,ti.	26030
#29	highschool*.ab,ti.	38
#30	"primary school*".ab,ti.	10008
#31	"secondary school*".ab,ti.	8411
#32	daycare.ab,ti.	1089
#33	teen.ab,ti.	4542
#34	teens.ab,ti.	5401
#35	teenage*.ab,ti.	18506
#36	"child" [Mesh]	1909942
#37	"adolescent" [Mesh]	1860873
#38	"infant" [Mesh]	1099343
**#39**	**or/1-38**	4031410

Factors

#40	risk*.ab,ti.	1774163
#41	"risk factor*".ab,ti.	471078

#42	“protective factor*”.ab,ti.	13206
#43	“promotive factor*”.ab,ti.	87
#44	predict*.ab,ti.	1261638
#45	likelihood.ab,ti.	113760
#46	forecast*.ab,ti.	12844
#47	associat*.ab,ti.	3653981
#48	correlat*.ab,ti.	1578641
#49	covariate*.ab,ti.	46904
#50	covariance.ab,ti.	18904
#51	pathway*.ab,ti.	896367
#52	“risk factors” [Mesh]	870976
#53	“protective factors” [Mesh]	8969
**#54**	**or/40-53**	7183780

Violence

#55	(physical adj2 violen*).ab,ti.	2263
#56	(sexual adj2 violen*).ab,ti.	2996
#57	(emotional adj2 violen*).ab,ti.	261
#58	(psychological adj2 violen*).ab,ti.	539
#59	(mental adj2 violen*).ab,ti.	449
#60	(physical adj2 abuse).ab,ti.	5027
#61	(sexual adj2 abuse).ab,ti.	11134
#62	(emotional adj2 abuse).ab,ti.	1660

#63	(mental adj2 abuse).ab,ti.	1104
#64	(psychological adj2 abuse).ab,ti.	766
#65	(verbal adj2 abuse).ab,ti.	718
#66	“child abuse”.ab,ti.	7672
#67	(physical adj2 assault*).ab,ti.	906
#68	(sexual adj2 assault*).ab,ti.	3936
#69	“physical attack*”.ab,ti.	141
#70	(severe adj2 punishment).ab,ti.	81
#71	(harsh adj2 punishment).ab,ti.	85
#72	“corporal punishment”.ab,ti.	428
#73	“corporeal punishment”.ab,ti.	4
#74	(severe adj2 discipline).ab,ti.	11
#75	(harsh adj2 discipline).ab,ti.	154
#76	“harsh parenting”.ab,ti.	223
#77	harass*ab,ti.	2828
#78	incest.ab,ti.	1266
#79	maltreat*.ab,ti.	5614
#80	“sexual violation*”.ab,ti.	36
#81	“forced sex”.ab,ti.	302
#82	“coerced sex”.ab,ti.	37
#83	rape.ab,ti.	6412
#84	mistreat*.ab,ti.	1452
#85	molest*.ab,ti.	1134

#86	defile*.ab,ti.	57
#87	(adverse adj childhood adj experience*).ab,ti.	794
#88	“ACE”.ab,ti.	30565
#89	“violence against children”.ab,ti.	255
#90	“rape" [Mesh]	9787
#91	“child abuse” [Mesh]	30306
#92	**or/55-91**	88996
**Lower and middle-income countries**		
#93	africa.ti,ab.	88138
#94	asia.ti,ab.	46392
#95	"west indies".ti,ab.	2179
#96	caribbean.ti,ab.	11373
#97	"south america".ti,ab.	11415
#98	"latin america".ti,ab.	11124
#99	"central america".ti,ab.	3211
#100	“middle east”.ti,ab.	8237
#101	“eastern Europe”.ti,ab.	4441
#102	“western pacific”.ti,ab.	1666
#103	(asia adj2 pacific).ti,ab.	3000
#104	“pacific island*”.ti,ab.	4590
#105	“polynesia".ti,ab.	1092

#106	“south pacific”,ti.ab.	1226
#107	“south sea island*”.ti,ab.	11
#108	oceana.ti,ab.	21
#109	“eastern mediterranean”.ti,ab.	1722
#110	Afghanistan.ti,ab.	4705
#111	Guinea.ti,ab.	103108
#112	Rwanda.ti,ab.	2128
#113	Benin.ti,ab.	2765
#114	Guinea-Bissau.ti,ab.	846
#115	Senegal.ti,ab.	4864
#116	“Burkina Faso”.ti,ab.	3124
#117	Haiti.ti,ab.	2522
#118	“Sierra Leone”.ti,ab.	1714
#119	Burundi.ti,ab.	674
#120	Korea.ti,ab.	33644
#121	Somalia.ti,ab.	1134
#122	"Central Africa Republic".ti,ab.	11
#123	Liberia.ti,ab.	1259
#124	"South Sudan".ti,ab.	349
#125	Chad.ti,ab.	952
#126	Madagascar.ti,ab.	3927
#127	Tanzania.ti,ab.	9963
#128	Comoros.ti,ab.	265

#129	Malawi.ti,ab.	5175
#130	Togo.ti,ab.	1196
#131	Congo.ti,ab.	9546
#132	Mali.ti,ab.	2836
#133	Uganda.ti,ab.	10905
#134	Eritrea.ti,ab.	410
#135	Mozambique.ti,ab.	2674
#136	Zimbabwe.ti,ab.	4585
#137	Ethiopia.ti,ab.	10159
#138	Nepal.ti,ab.	7253
#139	Gambia.ti,ab.	2040
#140	Niger.ti,ab.	10588
#141	Armenia.ti,ab.	822
#142	Kiribati.ti,ab.	145
#143	“Solomon Islands".ti,ab.	676
#144	Bangladesh.ti,ab.	10272
#145	Kosovo.ti,ab.	764
#146	“Sri Lanka”.ti,ab.	5013
#147	Bhutan.ti,ab.	494
#148	"Kyrgyz Republic".ti,ab.	46
#149	Kyrgyzstan.ti,ab.	406
#150	Sudan.ti,ab.	6741
#151	Bolivia.ti,ab.	2675

#152	"Lao PDR".ti,ab.	635
#153	Laos.ti,ab.	1433
#154	Swaziland.ti,ab.	696
#155	“Cabo Verde”.ti,ab.	58
#156	"Cape Verde".ti,ab.	439
#157	Lesotho.ti,ab.	545
#158	"Syrian Arab Republic".ti,ab.	68
#159	Syria.ti,ab.	1372
#160	Cambodia.ti,ab.	2981
#161	Mauritania.ti,ab.	509
#162	Tajikistan.ti,ab.	438
#163	Cameroon.ti,ab.	5462
#164	Micronesia.ti,ab.	566
#165	Timor-Leste.ti,ab.	230
#166	Moldova.ti,ab.	388
#167	Tonga.ti,ab.	382
#168	“cote d’ivoire”.ti,ab.	1780
#169	"Ivory Coast".ti,ab.	1589
#170	Mongolia.ti,ab.	3011
#171	Tunisia.ti,ab.	5371
#172	Djibouti.ti,ab.	316
#173	Morocco.ti,ab.	4411
#174	Ukraine.ti,ab.	3752

#175	Egypt.ti,ab.	11296
#176	Myanmar.ti,ab.	2149
#177	Uzbekistan.ti,ab.	977
#178	"El Salvador".ti,ab.	1061
#179	Nicaragua.ti,ab.	1552
#180	Vanuatu.ti,ab.	542
#181	Ghana.ti,ab.	7424
#182	Nigeria.ti,ab.	22938
#183	Vietnam.ti,ab.	11167
#184	Guatemala.ti,ab.	2867
#185	Pakistan.ti,ab.	13276
#186	"West Bank".ti,ab.	513
#187	Gaza.ti,ab.	799
#188	Honduras.ti,ab.	1407
#189	"Papua New Guinea".ti,ab.	4038
#190	Yemen.ti,ab.	1509
#191	India.ti,ab.	78332
#192	Philippines.ti,ab.	6722
#193	Zambia.ti,ab.	4095
#194	Indonesia.ti,ab.	8947
#195	Samoa.ti,ab.	754
#196	Kenya.ti,ab.	14162
#197	"Sao Tome".ti,ab.	213

#198	Principe.ti,ab.	260
#199	Albania.ti,ab.	855
#200	Ecuador.ti,ab.	3355
#201	Montenegro.ti,ab.	669
#202	Algeria.ti,ab.	2533
#203	Fiji.ti,ab.	1324
#204	Namibia.ti,ab.	1164
#205	Gabon.ti,ab.	1495
#206	Palau.ti,ab.	313
#207	Angola.ti,ab.	1118
#208	Georgia.ti,ab.	8173
#209	Panama.ti,ab.	3412
#210	Argentina.ti,ab.	13254
#211	Grenada.ti,ab.	256
#212	Paraguay.ti,ab.	1178
#213	Azerbaijan.ti,ab.	1083
#214	Guyana.ti,ab.	809
#215	Peru.ti,ab.	8140
#216	Belarus.ti,ab.	1090
#217	Iran.ti,ab.	27117
#218	Romania.ti,ab.	4337
#219	Belize.ti,ab.	669
#220	Iraq.ti,ab.	5485

#221	"Russian Federation".ti,ab.	2928
#222	Russia.ti,ab.	12993
#223	Bosnia.ti,ab.	1926
#224	Herzegovina.ti,ab.	1441
#225	Jamaica.ti,ab.	2913
#226	Serbia.ti,ab.	3405
#227	Botswana.ti,ab.	1827
#228	Jordan.ti,ab.	4616
#229	"South Africa".ti,ab.	25899
#230	Brazil.ti,ab.	65419
#231	Kazakhstan.ti,ab.	1666
#232	"St. Lucia".ti,ab.	257
#233	"Saint Lucia".ti,ab.	59
#234	Bulgaria.ti,ab.	3591
#235	Lebanon.ti,ab.	3352
#236	"St. Vincent".ti,ab.	386
#237	"Saint Vincent".ti,ab.	167
#238	Grenadines.ti,ab.	57
#239	China.ti,ab.	121025
#240	Libya.ti,ab.	1063
#241	Suriname.ti,ab.	441
#242	Colombia.ti,ab.	9130
#243	Macedonia.ti,ab.	943

#244	Thailand.ti,ab.	21830
#245	"Costa Rica".ti,ab.	4048
#246	Malaysia.ti,ab.	12041
#247	Turkey.ti,ab.	29359
#248	Cuba.ti,ab.	3914
#249	Maldives.ti,ab.	231
#250	Turkmenistan.ti,ab.	276
#251	Dominica.ti,ab.	380
#252	"Marshall Islands".ti,ab.	260
#253	Tuvalu.ti,ab.	51
#254	"Dominican Republic".ti,ab.	1530 808
#255	Mauritius.ti,ab.	808
#256	Venezuela.ti,ab.	4636
#257	"Equatorial Guinea".ti,ab.	346
#258	Mexico.ti,ab.	34201
#259	Cameroon.ti,ab.	5462
#260	Hungary.ti,ab.	8995
#261	Seychelles.ti,ab.	587
#262	Uruguay.ti,ab.	2223
#263	"Saint Kitts".ti,ab.	18
#264	"St. Kitts".ti,ab.	150
#265	Nevis.ti,ab.	91
#266	Poland.ti,ab.	19583

#267	Lithuania.ti,ab.	2079
#268	Latvia.ti,ab.	1052
#269	Croatia.ti,ab.	5264
#270	Chile.ti,ab.	10628
#271	Antigua.ti,ab.	198
#272	Barbuda.ti,ab.	53
#273	Aruba.ti,ab.	149
#274	Bahrain.ti,ab.	722
#275	Barbados.ti,ab.	863
#276	"Czech Republic".ti,ab.	6554
#277	Czechoslovakia.ti,ab.	3116
#278	Estonia.ti,ab.	2121
#279	Gibraltar.ti,ab.	285
#280	Greece.ti,ab.	12104
#281	Guam.ti,ab.	1049
#282	“Isle of Man”.ti,ab.	118
#283	Macao.ti,ab.	243
#284	"New Caledonia".ti,ab.	1252
#285	"Northern Mariana Islands".ti,ab.	106
#286	Oman.ti,ab.	2113
#287	Portugal.ti,ab.	9296
#288	"Puerto Rico".ti,ab.	5409
#289	"Saudi Arabia".ti,ab.	12003

#290	Slovakia.ti,ab.	3007
#291	"Slovak Republic".ti,ab.	580
#292	Slovenia.ti,ab.	2676
#293	Trinidad.ti,ab.	1963
#294	Tobago.ti,ab.	776
#295	Mayotte.ti,ab.	228
#296	"Netherlands Antilles".ti,ab.	147
#297	USSR.ti,ab.	6768
#298	"Soviet Union".ti,ab.	3410
#299	"Soviet Socialist Republics".ti,ab.	88
#300	Yugoslavia.ti,ab.	2238
#301	"developing countr*".ti,ab.	50127
#302	"developing nation*".ti,ab.	2442
#303	"developing population*".ti,ab.	283
#304	"developing world".ti,ab.	7388
#305	"developing econom*".ti,ab.	363
#306	"low income countr*".ti,ab.	4567
#307	"middle income countr*".ti,ab.	9918
#308	"low income nation*".ti,ab.	63
#309	"middle income nation*".ti,ab.	71
#310	LMIC.ti,ab.	867
#311	LMICS.ti,ab.	1581
#312	"third world".ti,ab.	2928

#313	"LAMI countr*".ti,ab.	36
#314	"transitional countr*".ti,ab.	139
#315	“developing countries” [Mesh]	109580
#316	**or/93-315**	1115733
#317	39 and 54 and 92 and 316	2585
#318	Limit 317 to (english language and humans and yr=”1989-current”)	1968
**#319**	**318 not ((case report or editorial).ti,ab. or editorial.ptyp. or letter.ptyp. or newspaper article.ptyp.)**	1965
