# Supplementary material for: Factors Associated With Violence Against Children in Low- and Middle-Income Countries: A Systematic Review and Meta-Regression of Nationally Representative Data
Source: Trauma Violence Abuse. 2021 Jan 19;22(2):219–32. doi: 10.1177/1524838020985532 (PMC7961628; doi:10.1177/1524838020985532)
Supplement: Supplemental Material, Appendix_B - Factors Associated With Violence Against Children in Low- and Middle-Income Countries: A Systematic Review and Meta-Regression of Nationally Representative Data [file Appendix_B.pdf]

Appendix B. List of included databases

Name	
1	Medline/PubMed
2	Embase
3	PsychINFO
4	Global Health
5	Social Policy and Practice
6	Scopus
7	CINAHL Plus
8	Africa-Wide Information
9	Cochrane Systematic Reviews
10	Web of Science
11	IBSS
12	LILACS
13	MedCarib
14	IMEMR
15	WPRIM
16	IndMED
17	CNKI English
