# Supplementary material for: Factors Associated With Violence Against Children in Low- and Middle-Income Countries: A Systematic Review and Meta-Regression of Nationally Representative Data
Source: Trauma Violence Abuse. 2021 Jan 19;22(2):219–32. doi: 10.1177/1524838020985532 (PMC7961628; doi:10.1177/1524838020985532)
Supplement: Supplemental Material, Appendix_C - Factors Associated With Violence Against Children in Low- and Middle-Income Countries: A Systematic Review and Meta-Regression of Nationally Representative Data [file Appendix_C.pdf]

Appendix C. Identified factors and measures by physical, emotional, and sexual violence types

Violence types	Factors and measures identified in the studies	Factors and measures transformed for data synthesis	Final factors included in the meta-regression
**1) Physical**	• **Absent father**<br>- Yes, no	• **Absent father**<br>- No, yes	1. Adult in household education level – No education<br>2. Adult in household education level – Primary level<br>3. Child labor<br>4. Child gender<br>5. Household wealth – First quintile<br>6. Household wealth – Second quintile
	• **Absent mother**<br>- Yes, no	• **Absent mother**<br>- No, yes	
	• **Adult in household education level**<br>- No education, basic/pre-primary, primary, some primary, secondary, some secondary, lower secondary, upper secondary, tertiary, post-graduate	• **Adult in household education level**<br>- Tertiary level or above, secondary level, primary level, no education	

	- Non-standard curriculum<br>- Specialized secondary/vocational<br>- None, middle, higher or university		7. Household wealth – Third quintile<br>8. Household wealth – Fourth quintile<br>9. Mother education level – No education<br>10. Mother education level – Primary education<br>11. Mother experienced any form of intimate partner violence (IPV) – Yes<br>12. Rural residence
	• **Child age**<br>- 0-17 years - continuous<br>- 0-17 years - variable categorical age blocks	• **Child age**<br>- 13-17, 6-12, 0-5 years	
	• **Child disability**<br>- Yes, no	• **Child disability**<br>- No, yes	
	• **Child ever worked as domestic servant**<br>- Yes, no<br>• **Child ever worked for money or other payments**	• **Child labor**<br>- No, yes	

	- Yes, no		
	• **Child gender**<br>- Boys, girls	• **Child gender**<br>- Boys, girls	
	• **Child past experience of physical violence**<br>- Yes, no	• **Child past experience of physical violence**<br>- No, yes	
	• **Child past self-harm**<br>- Yes, no	• **Child past self-harm**<br>- No, yes	
	• **Child past suicide attempt**<br>- Yes, no	• **Child past suicide attempt**<br>- No, yes	
	• **Child perpetration of violence at any point**<br>- Yes, no	• **Child perpetration of violence**<br>- No, yes	

	• **Child perpetration of physical or sexual violence in the past 12 months**<br>- Yes, no		
	• **Community average access to abortion**<br>- Continuous<br>• **Community average access to prenatal care**<br>- Continuous	• **Community average access to health services**<br>- Continuous	
	• **Country average amount of armed conflict**<br>- Continuous	• **Country average amount of armed conflict**<br>- Continuous	
	• **Community average economic freedom**<br>- Continuous	• **Community economic indicators**<br>- Continuous	

	• **Community average economic growth**<br>- Continuous<br>• **Community average Gross Domestic Product (GDP) per capita**<br>- Continuous<br>• **Community average income inequality**<br>- Continuous<br>• **Community average poverty levels**<br>- Continuous<br>• **Community economic development index**<br>- Continuous		

	• **Community Human Development Index (HDI)**<br>- Continuous		
	• **Community average life expectancy**<br>- Continuous	• **Community average life expectancy**<br>- Continuous	
	• **Community average schooling levels**<br>- Continuous<br>• **Community education index**<br>- Continuous<br>• **Community literacy index**<br>- Continuous	• **Community average schooling levels**<br>- Continuous	
	• **Community average schooling levels for women**<br>- Continuous	• **Community gender inequality indicators**<br>- Continuous	

	• **Community average weeks of maternal leave**<br>- Continuous<br>• **Community gender inequality index**<br>- Continuous<br>• **Community Social, Institutional, and Gender index (SIGI)**<br>- Continuous		
	• **Double orphan status**<br>- Yes, no<br>• **Single orphan status**<br>- Yes, no	• **Single or double orphan status**<br>- No, yes	
	• **Father employment grade**<br>- Professional, managerial, or administrative, agricultural	• **Father employment grade**<br>- Professional/office work, non-professional, unemployed	

	sector, domestic work, sales, unemployed		
	• **Father experience of abuse as a child**<br>- Yes, no	• **Father experience of abuse as a child**<br>- No, yes	
	• **Gender of the household head**<br>- Man, woman	• **Gender of the household head**<br>- Man, woman	
	• **Household crowding**<br>- Less than 6, greater than 6<br>- Less than 5, greater than 5<br>- 1-3 per room, 4-10 per room<br>• **Number of children in household**<br>- 1, 2-3, 4 or more<br>- Average	• **Household crowding**<br>- No, yes	
	• **Household head education level**	• **Household head education level**	

	- No education, basic/pre-primary, primary, some primary, secondary, some secondary, lower secondary, upper secondary, tertiary, post-graduate<br>- Non-standard curriculum<br>- Specialized secondary/vocational<br>- None, middle, higher or university	- Tertiary level or above, secondary level, primary level, no education	
	• **Household wealth**<br>- Five wealth quintiles – richest to poorest<br>- 40% richest, 60% poorest<br>- 60% richest, 40% poorest	• **Household wealth**<br>- Five wealth quintiles - richest to poorest<br>- 60% richest, 40% poorest	

	- 20% poorest, 40% middle, 40% richest		
	• **Malnutrition**<br>- Continuous	• **Malnutrition**<br>- Continuous	
	• **Marital status of child**<br>- Never married, married or in a marriage like relationship	• **Marital status of child**<br>- Never married, married or in a marriage like relationship	
	• **Marital status of parents**<br>- Married or in a marriage like relationship, previously married, never married	• **Marital status of parents**<br>- Married or in a marriage like relationship, previously married, never married	
	• **Maternal age**<br>- 15-49 years – continuous<br>- 15-49 years – variable categorical age blocks	• **Maternal age**<br>- Older than 35, 25-35, 16-24 years	

	• **Mother education level**<br>- No education, basic/pre-primary, primary, some primary, secondary, some secondary, lower secondary, upper secondary, tertiary, post-graduate<br>- Non-standard curriculum<br>- Specialized secondary/vocational<br>- None, middle, higher or university	• **Mother education level**<br>- Tertiary level or above, secondary level, primary level, no education	
	• **Mother employment status**<br>- Employed, unemployed	• **Mother employment status**<br>- Employed, unemployed	
	• **Mother experience of abuse as child**	• **Mother experience of abuse as child**	

	- Yes, no	- No, yes	
	• **Mother experienced any form of intimate partner violence (IPV)**<br>- Yes, no<br>- Continuous IPV experience score 0-7<br>- Frequently, sometimes, never	• **Mother experienced any form of intimate partner violence (IPV)**<br>- No, yes<br>- Continuous IPV experience score 0-7	
	• **Number of adult women in household**<br>- Continuous	• **Number of adult women in household**<br>- Continuous	
	• **Parental age**<br>- 16-24, 25-35, older than 35 years	• **Parental age**<br>- Older than 35, 25-35, 16-24 years	
	• **Parental disability**<br>- Yes, no	• **Parental disability**<br>- No, yes	

	• **Parental mental health**<br>- Mean – self-reporting questionnaire-20	• **Parental mental health**<br>- Mean – self-reporting questionnaire-20	
	• **Parental substance abuse**<br>- Yes, no	• **Parental substance abuse**<br>- No, yes	
	• **Paternal age**<br>- 16-24, 25-35, older than 35 years	• **Paternal age**<br>- Older than 35 years, 25-35, 16-24 years	
	• **Place of residence**<br>- Urban, rural, camps<br>- Urban, rural without road, rural with road<br>- Urban, rural coastal, rural interior	• **Place of residence**<br>- Urban, rural, camps	
	• **Policies legitimizing the death penalty or capital punishment**	• **Policies legitimizing the death penalty or capital punishment**	

	- Above average, below average	- Above average, below average	
	• **Traded sex for goods or money**<br>- Yes, no	• **Traded sex for goods or money**<br>- No, yes	

A list of factors identified in the studies and how they were measured is included in the second column. The categories created for analysis purposes are found in the third column. The research team attempted to maintain categories that were as close as possible to the original factors and measurements found in the studies when creating cross-comparable categories. The reference group is the first measurement that appears after each factor within the third column. The list of the factors with a sufficient number of estimates to be analyzed as part of the meta-regression are included in the fourth column.

Violence types	Factors and measures identified in the studies	Factors and measures transformed for data synthesis	Final factors included in the meta-regression
**2) Emotional**	• **Absent father**<br>- Yes, no	• **Absent father**<br>- No, yes	1. Adult in household education level – No education<br>2. Adult in household education level – Primary level<br>3. Child gender<br>4. Household wealth – First quintile<br>5. Household wealth – Second quintile<br>6. Household wealth – Third quintile
	• **Absent mother**<br>- Yes, no	• **Absent mother**<br>- No, yes	
	• **Adult in household education level**<br>- No education, basic/pre-primary, primary, some primary, secondary, some secondary, lower secondary, upper secondary, tertiary, post-graduate<br>- Non-standard curriculum	• **Adult in household education level**<br>- Tertiary level or above, secondary level, primary level, no education	

	- Specialized secondary/vocational<br>- None, middle, higher or university		7. Household wealth – Fourth quintile<br>8. Mother education level – No education<br>9. Mother education level – Primary level<br>10. Rural residence
	• **Child age**<br>- 0-17 years - continuous<br>- 0-17 years - variable categorical age blocks	• **Child age**<br>- 13-17, 6-12, 0-5 years	
	• **Child disability**<br>- Yes, no	• **Child disability**<br>- No, yes	
	• **Child ever worked as domestic servant**<br>- Yes, no<br>• **Child ever worked for money or other payments**<br>- Yes, no	• **Child labor**<br>- No, yes	

	• **Child gender**<br>- Boys, girls	• **Child gender**<br>- Boys, girls	
	• **Child past experience of emotional violence**<br>- Yes, no<br>• **Child past experience of physical violence**<br>- Yes, no<br>• **Child past experience of sexual violence**<br>- Yes, no	• **Child past experience of any form of violence**<br>- No, yes	
	• **Child past self-harm**<br>- Yes, no	• **Child past self-harm**<br>- No, yes	
	• **Child past suicide attempt**<br>- Yes, no	• **Child past suicide attempt**<br>- No, yes	

	• **Community average Gross Domestic Product (GDP) per capita**<br>- Continuous<br>• **Community economic development index**<br>- Continuous<br>• **Community Human Development Index (HDI)**<br>- Continuous	• **Community economic indicators**<br>- Continuous	
	• **Community average schooling levels**<br>- Continuous<br>• **Community education index**<br>- Continuous<br>• **Community literacy index**	• **Community education indicators**<br>- Continuous	

	- Continuous		
	• **Community average life expectancy**<br>- Continuous	• **Community average life expectancy**<br>- Continuous	
	• **Double orphan status**<br>- Yes, no<br>• **Single orphan status**<br>- Yes, no	• **Single or double orphan status**<br>- No, yes	
	• **Father employment grade**<br>- Professional, managerial, or administrative, agricultural sector, domestic work, sales, unemployed	• **Father employment grade**<br>- Professional/office work, non-professional, unemployed	
	• **Father experience of abuse as a child**	• **Father experience of abuse as a child**	

	- Yes, no	- No, yes	
	• **Food security**<br>- Food in house last week, food not in house last week	• **Food security**<br>- Food in house last week, food not in house last week	
	• **Gender of the household head**<br>- Man, woman	• **Gender of the household head**<br>- Man, woman	
	• **Household crowding**<br>- Less than 6, greater than 6<br>- Less than 5, greater than 5<br>- 1-3 per room, 4-10 per room<br>• **Number of children in household**<br>- 1, 2-3, 4 or more<br>- Average	• **Household crowding**<br>- No, yes	

	• **Household head education level**<br>- No education, basic/pre-primary, primary, some primary, secondary, some secondary, lower secondary, upper secondary, tertiary, post-graduate<br>- Non-standard curriculum<br>- Specialized secondary/vocational<br>- None, middle, higher or university	• **Household head education level**<br>- Tertiary level or above, secondary level, primary level, no education	
	• **Household instability**<br>- Moved less than three times, moved more than three times	• **Household instability**<br>- Moved less than three times, moved more than three times	

	• **Household wealth**<br>- Five wealth quintiles – richest to poorest<br>- 40% richest, 60% poorest<br>- 60% richest, 40% poorest<br>- 20% poorest, 40% middle, 40% richest	• **Household wealth**<br>- Five wealth quintiles - richest to poorest<br>- 60% richest, 40% poorest	
	• **Marital status of child**<br>- Never married, married or in a marriage like relationship	• **Marital status of child**<br>- Never married, married or in a marriage like relationship	
	• **Maternal age**<br>- 15-49 years – variable categorical age blocks	• **Maternal age**<br>- Older than 35, 25-35, 16-24 years	
	• **Mother education level**<br>- No education, basic/pre-primary, primary, some	• **Mother education level**	

	primary, secondary, some secondary, lower secondary, upper secondary, tertiary, post-graduate<br>- Non-standard curriculum<br>- Specialized secondary/vocational<br>- None, middle, higher or university	- Tertiary level or above, secondary level, primary level, no education	
	• **Mother employment status**<br>- Employed, unemployed	• **Mother employment status**<br>- Employed, unemployed	
	• **Mother experience of abuse as child**<br>- Yes, no	• **Mother experience of abuse as child**<br>- No, yes	

	• **Mother experienced any form of intimate partner violence (IPV)**<br>- Yes, no	• **Mother experienced any form of intimate partner violence (IPV)**<br>- No, yes	
	• **Parental disability**<br>- Yes, no	• **Parental disability**<br>- No, yes	
	• **Place of residence**<br>- Urban, rural, camps<br>- Urban, rural without road, rural with road<br>- Urban, rural coastal, rural interior	• **Place of residence**<br>- Urban, rural, camps	
	• **Quality of the parental relationship**<br>- Close to parent, not close to parent	• **Quality of the parental relationship**<br>- Close to parent, not close to parent	

	• **Traded sex for goods or money**<br>- Yes, no	• **Traded sex for goods or money**<br>- No, yes	

A list of factors identified in the studies and how they were measured is included in the second column. The categories created for analysis purposes are found in the third column. The research team attempted to maintain categories that were as close as possible to the original factors and measurements found in the studies when creating cross-comparable categories. The reference group is the first measurement that appears after each factor within the third column. The list of the factors with a sufficient number of estimates to be analyzed as part of the meta-regression are included in the fourth column.

Violence types	Factors and measures identified in the studies	Factors and measures transformed for data synthesis	Final factors included in the meta-regression
**3) Sexual**	• **Absent father**<br>- Yes, no	• **Absent father**<br>- No, yes	
	• **Absent mother**<br>- Yes, no	• **Absent mother**<br>- No, yes	
	• **Absent parent**<br>- Yes, no	• **Absent parent**<br>- No, yes	
	• **Child age**<br>- 0-17 years - continuous<br>- 0-17 years - variable categorical age blocks	• **Child age**<br>- 13-17, 6-12, 0-5 years	
	• **Child disability**<br>- Yes, no	• **Child disability**<br>- No, yes	

	• **Child ever worked as domestic servant**<br>- Yes, no<br>• **Child ever worked for money or other payments**<br>- Yes, no	• **Child labor**<br>- No, yes	
	• **Child gender**<br>- Boys, girls	• **Child gender**<br>- Boys, girls	
	• **Child knowledge of rights**<br>- Above national average, below national average	• **Child knowledge of rights**<br>- Above national average, below national average	
	• **Child level of education**<br>- Completed less than primary, completed more than primary	• **Child level of education**<br>- Completed more than primary, completed less than primary	

	• **Child past experience of emotional violence**<br>- Yes, no<br>• **Child past experience of physical violence**<br>- Yes, no<br>• **Child past experience of sexual violence**<br>- Yes, no	• **Child past experience of any form of violence**<br>- No, yes	
	• **Child past self-harm**<br>- Yes, no	• **Child past self-harm**<br>- No, yes	
	• **Child past suicide attempt**<br>- Yes, no	• **Child past suicide attempt**<br>- No, yes	
	• **Child sexual debut**<br>- Yes, no	• **Child sexual debut**<br>- Yes, no	

	• **Community levels of intimate partner violence (IPV) in past year**<br>- Below national average, above national average	• **Community levels of intimate partner violence (IPV) in past year**<br>- Below national average, above national average	
	• **Double orphan status**<br>- Yes, no<br>• **Single orphan status**<br>- Yes, no	• **Single or double orphan status**<br>- No, yes	
	• **Food security**<br>- Food in house last week, food not in house last week	• **Food security**<br>- Food in house last week, food not in house last week	
	• **HIV prevention programs at the national level**<br>- Yes, no	• **HIV prevention programs at the national level**<br>- Yes, no	

	• **Household crowding**<br>- 1-3 per room, 4-10 per room	• **Household crowding**<br>- No, yes	
	• **Marital status of child**<br>- Never married, married or in a marriage like relationship	• **Marital status of child**<br>- Never married, married or in a marriage like relationship	
	• **Parental knowledge of sexual risk**<br>- Yes, no	• **Parental knowledge of sexual risk**<br>- Yes, no	
	• **Parental substance abuse**<br>- Yes, no	• **Parental substance abuse**<br>- No, yes	
	• **Place of residence**<br>- Urban, rural	• **Place of residence**<br>- Urban, rural	
	• **Quality of the parental relationship**<br>- Close to parent, not close to parent	• **Quality of the parental relationship**<br>- Close to parent, not close to parent	

	• School level perpetration of sexual violence<br>- Below national average, above national average	• School level perpetration of sexual violence<br>- Below national average, above national average	
	• School levels of substance use<br>- Below national average, above national average	• School levels of substance use<br>- Below national average, above national average	
	• Traded sex for goods or money<br>- Yes, no	• Traded sex for goods or money<br>- Yes, no	

A list of factors identified in the studies and how they were measured is included in the second column. The categories created for analysis purposes are found in the third column. The research team attempted to maintain categories that were as close as possible to the original factors and measurements found in the studies when creating cross-comparable categories. The reference group is the first measurement that appears after each factor within the third column. The list of the factors with a sufficient number of estimates to be analyzed as part of the meta-regression are included in the fourth column.
