# Supplementary material for: Factors Associated With Violence Against Children in Low- and Middle-Income Countries: A Systematic Review and Meta-Regression of Nationally Representative Data
Source: Trauma Violence Abuse. 2021 Jan 19;22(2):219–32. doi: 10.1177/1524838020985532 (PMC7961628; doi:10.1177/1524838020985532)
Supplement: Supplemental Material, Appendix_D - Factors Associated With Violence Against Children in Low- and Middle-Income Countries: A Systematic Review and Meta-Regression of Nationally Representative Data [file Appendix_D.pdf]

Appendix D. *p*-value plots for publication bias

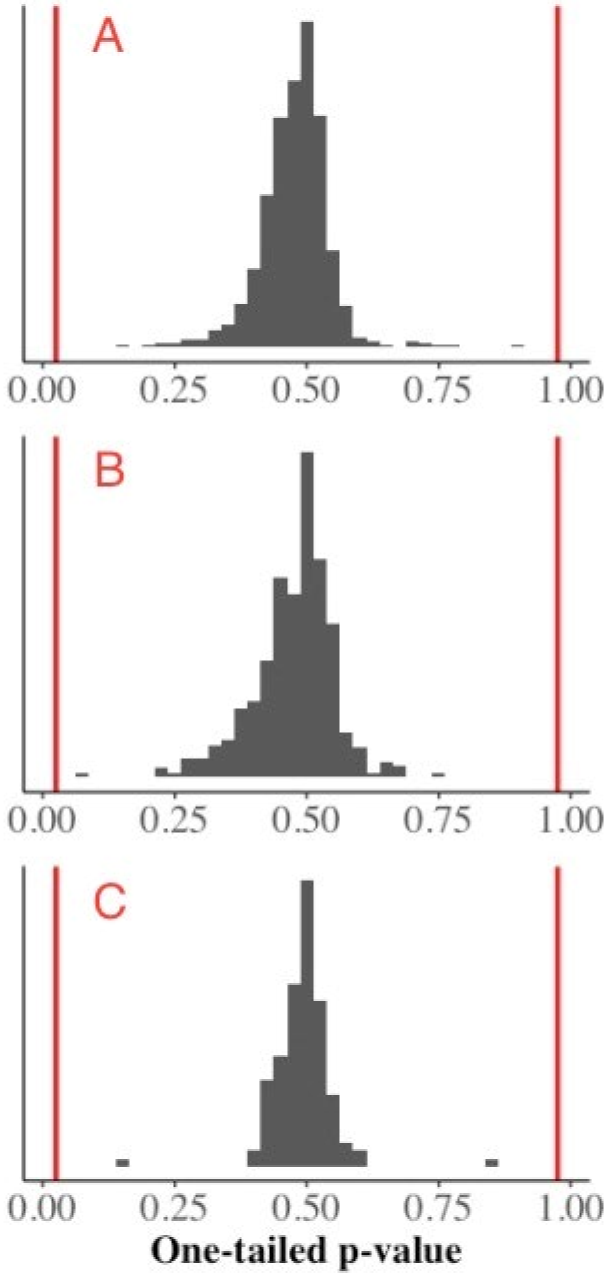

Figure A - physical violence; Figure B - emotional violence; Figure C - sexual violence.

The red lines indicate the extreme values of the tails of the distribution (upper 5%) in which it is assumed that positive results would be favored, leading to possible publication bias. Concentration of studies to the right of the upper red line suggest two-sided tests are appropriate (Mathur & VanderWeele, 2020). All studies fall within the middle 95% of the distribution for violence outcomes in the three figures.
